# Can Mindfulness-Based Stress Reduction Influence the Quality of Life, Anxiety, and Depression of Women Diagnosed with Breast Cancer? —A Review

**DOI:** 10.3390/curroncol29100615

**Published:** 2022-10-15

**Authors:** Severin Ladenbauer, Josef Singer

**Affiliations:** 1 Karl Landsteiner University of Health Sciences, 3500 Krems, Austria; 2 Department of Internal Medicine 2, University Hospital Krems, 3500 Krems, Austria

**Keywords:** mindfulness-based stress reduction, breast cancer, quality of life, anxiety, depression

## Abstract

Introduction: Breast cancer is the most common form of cancerous disease worldwide. Its treatment leads to a variety of physiological and psychological side effects. This review investigates the question of how mindfulness-based stress reduction (MBSR), a stress management program, can influence the quality of life, anxiety, and depression of women diagnosed with breast cancer. Methods: A systematic literature search was conducted in PubMed/MEDLINE and Cochrane Library. Screening by title, abstract and full text was performed, whereby only those articles were included that fit the inclusion criteria. A risk of bias assessment was performed for each included study. Results: Overall, six studies were included, but not every study investigated all three outcomes. Two studies found positive impacts on quality of life, whereas three did not find a positive correlation between the intervention and quality of life. Four out of six studies found a positive relation between MBSR and anxiety scores, but only half of the included studies found positive results for the interaction between MBSR and depression scores. Conclusion: Published data suggest that anxiety can be positively influenced by MBSR, which can be used to improve the psychological care of breast cancer patients, both during and after treatment. However, further studies with larger patient numbers and longer observation periods should be conducted in order to elucidate the full potential of MSBR on important areas such as depression and quality of life.

## 1. Introduction

Due to the severity of the disease, a high percentage of women diagnosed with breast cancer develop psychological problems during or after their treatment. The most frequent symptoms are fatigue, increased psychological stress, anxiety, and depression, along with the fear of recurrence or worsening of the disease [1].

Quality of life can deteriorate through somatic and psychological side effects of both the illness itself, and the different forms of treatment. The mental burden that is imposed on breast cancer patients poses a high need for interventions, which tackle these side effects and their physiological consequences [1].

The practice of mindfulness-based stress reduction (MBSR) was developed by Jon Kabat-Zinn, PhD in 1979. While Kabat-Zinn was lecturing at the University of Massachusetts, he founded the “Centre of mindfulness in Medicine, Health care and Society”. At the beginning, his program was referred to as the “Stress Reduction and Relaxation Program” and, although it was remarkably similar to MBSR nowadays, it was heavily rooted in Buddhist principles of mindfulness and meditation. Later on, along with a change of name, the program evolved from the original Buddhist mindset to rebuilding itself on a more secular foundation as it is known for today [2].

The principle of mindfulness is described as the ability to gain greater awareness of every moment without judgement over it. As part of the mindfulness-based stress reduction program, it is also described as deep immersion in one’s own being, in the spirit of self-reflection and self-discovery. If mindfulness is achieved, it can help the person see their circumstances more clearly and to find alternatives and possibilities for improving health and quality of life. In addition, mindfulness can provide new energy in stressful and straining situations and can help to utilise the individual’s energy reserve capacities more wisely. Learning and practicing mindfulness is the key goal of every MBSR course [2].

Mindfulness, which according to MBSR training is based on seven qualities of inner attitude, can be achieved through meditation and is the main technique utilised throughout MBSR courses [2].

MBSR courses are minimum eight-week programs that utilise different techniques to increase the mindfulness of its participants. The majority of the techniques are a form of meditation connected to self-reflection and introspection. MBSR courses can be completed in over 720 clinics worldwide that employ certified MBSR trainers, or at home via a written transcript of the course’s syllabus [2]. If performed in one of the training centres, a guided session takes place almost every week, whereby the focus changes weekly between different topics of awareness, each with its own special training techniques, awareness-exercises, and home assignment. The home assignment often includes a technique called a “body scan”, which is a special form of meditation with the goal of achieving thorough body awareness [2].

### Aim

The aim of this review is to investigate the effects that MBSR can have on several psychological factors, including quality of life, depression, and anxiety in breast cancer patients. This systematic review includes studies from 2011 onwards. However, as Schell and Monsef conducted a thorough meta-analysis of this topic in 2018, this review is predominantly focused on findings that are dated 2018 or later and compares the relevant findings to the meta-analysis of Schell and Monsef [3].

The primary research question is:

“Can mindfulness-based stress reduction have a positive impact on the quality of life of women that have been diagnosed with breast cancer?”

The secondary research question is:

“What impact does mindfulness-based stress reduction have on anxiety and depression in women that have been diagnosed with breast cancer?”

## 2. Materials and Methods

In order to identify relevant articles, a literature search in PubMed/MEDLINE and Cochrane Library was conducted. Articles that fit the predefined inclusion and exclusion criteria (Table 1) were included in this systematic review.

Table 2 shows the search strategy utilised in PubMed/MEDLINE. Free-text searches in addition to medical subject headings were used to find the largest number studies possible.

**Figure 1:**
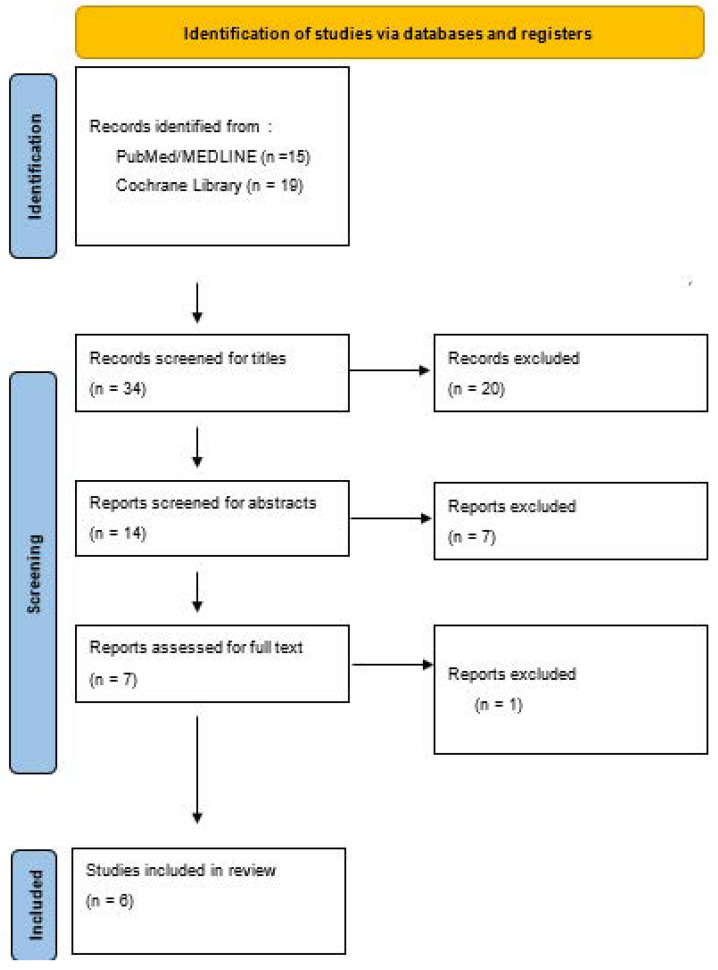
PRISMA Diagram of Screening Process.

The titles of the studies located were reviewed for eligibility against the a priori defined inclusion and exclusion criteria (Table 1), after which, an abstract analysis was performed, followed by a full text analysis. Only if all three (title, abstract, and full text analysis) matched the inclusion/exclusion criteria, the study was included in this review. Figure 1 shows this process as a Prisma Diagram.

All search results were imported and tracked in an EndNote^®^ bibliographic database.

For all studies that met the inclusion criteria, relevant and extracted information is provided in Table 3.

### 2.1. Risk of Bias Assessment

The risk of bias assessment for the randomised control trials was performed with the RoB 2 tool (a revised tool to asses risk of bias in randomised trials), and is summarised in Table 4 [10]. Table 4 is a modified version of the RoB 2 for better illustration.

The signalling questions were classified by “yes” (Y), “probably yes” (PY), “probably no” (PN), “no” (N), and “no information given” (NI). After each domain’s questions were answered, a risk of bias judgment concerning this subpart was surmised. These results led to the overall assessment of bias (“low risk”, “some concerns”, “high risk”).

The risk of bias assessment for non-randomised studies was performed with the ROBINS-I tool [10] and is shown as a modified version for better illustration below in Table 5.

The signalling questions were classified by “yes” (Y), “probably yes” (PY), “probably no” (PN), “no” (N), and “no information given” (NI). After each domain’s questions were answered, a risk of bias judgment concerning this subpart was surmised. These results led to an overall assessment of bias (“low risk”, “moderate”, “high risk”).

The two systematic reviews included by Chang et al. (2020) [8] and Zhang et al. (2018) [9] were assessed using the CASP (Critical Appraisal Skills Programme). All ten questions of the three sections of the CASP could be answered with sufficient accuracy for both publications.

### 2.2. Ethical Consideration

Because the study does not use individual patient data, no ethical approval was necessary.

### 2.3. Definition of Measurement Tools

The two tools assessing the quality of life used in the studies included: the 36-Item Medical Outcomes Studies Short-Form General Health Survey (36-SF) and the EuroQol 5-dimension health questionnaire (EQ-5D).

The 36-SF (Cronbach’s α 0.791) is a disease-unspecific tool for the survey of health-related quality of life. It consists of eight subcategories: physical functioning, physical role functioning, body pain, general health, vitality, social functioning, emotional role functioning, and mental health. By performing a factor analysis, those eight items can be explained with two dimensions, the mental component summaries (MCS) and physical component summaries (PCS), which account for 82% of the reliable variance of the measure [11]. The scores range 0–100, with a lower score corresponding to a higher disability and a higher score corresponding to a lower disability [12].

The EuroQol 5-dimension health questionnaire (EQ-5D) (Cronbach’s α > 0.7) is another measurement tool for the health-related quality of life. There are five subscales: mobility, self-care, everyday activity, pain and discomfort, and anxiety and depression. Patients provide accounts on each of these categories on a scale 0–100, with lower numbers corresponding to worse health [11].

The General Anxiety Questionnaire-7 (GAD7) (Cronbach’s α 0.92) is used for the identification of patients with possible anxiety disorders and to assess the symptoms and severity of existing anxiety in patients. It consists of seven self-rating questions, that include the various accompanying symptoms of an anxiety disorder [13]. Each question must be answered on a four-point scale, evaluating the frequency of the symptom in the last 14 days. Possible answers include: “not at all”, “several days”, “over half the time”, and “nearly every day”. Each answer correlates to a number (0–3) and the overall score is determined by adding all seven scores together (0–21). The result of the test depends on the overall score and can be classified into four categories (Table 6) [4].

The Patient Health Questionnaire-9 (PHQ9) (Cronbach’s α 0.85), which measures depression, is structured in the same way as the GAD7 (possible answers: “not at all”, “several days”, “over half the time”, “nearly every day”, correlating to a number 0–3), but uses nine questions in order to obtain an overall score as displayed in Table 7 [4].

The Depression, Anxiety and Stress Scale (DASS-21) (Cronbach’s α 0.82) is used to measure the overall mental state of a patient. It consists of three columns, each dedicated to one of the three factors, with 42 questions in total. Each question must be rated using a Likert-type scale 0–3. The score of each column can be analysed separately from the other two (Table 8) [5].

The Beck Anxiety Inventory (BAI) (Cronbach’s α 0.94) consists of 21 items and is used to assess symptoms of anxiety independently from symptoms of depression. Beck et al. [14] found somatic and cognitive-affective symptoms as dimensions in a factor analysis. Each of the 21 items must be rated on how much the patient is bothered by the item in everyday life, on a Likert-type scale from “not at all”, which results in zero, to “it bothers me a lot”, which results in three. The overall test score (Table 9) can be calculated by adding up all the results [15].

The Beck Depression Inventory-II (Cronbach’s α 0.89) (BDI-II) follows the organisation and scoring system of the BAI, but is used for the evaluation of depression [16].

The State-Trait Anxiety Inventory (STAI) consists of 20 items that must be rated on a Likert-type scale from “not at all”, which results in a score of one, to “very much so”, which results in a score of three. Calculation of the final test score provides information about the anxiety levels of the person, with higher scores meaning more anxiety [6].

The Centre for Epidemiological Studies Depression (CES-D) (Cronbach’s α 0.90) Scale is a self-report questionnaire, developed for the diagnosis of symptoms of depression. It consists of 20 items that must be rated on a Likert-scale from zero to three. The overall score, calculated by adding up all the individual ratings, determines whether the patient is suffering from depressive symptoms or not. At a score of more than 16, the patient is considered to have depressive symptoms [17].

## 3. Results

### 3.1. Primary Outcome: Quality of Life

The studies by Elimimian et al. (2020) [4], Lengacher et al. (2019) [6], Chang et al. (2022) [5], and the systematic reviews by Chang et al. (2020) [8] and Zhang et al. (2018) [9] were included in the primary outcome analysis. The study by Mirmahmoodi et al. (2020) [7] could not be included because it did not use an individual measurement tool for quality of life but rather used the term to summarise different effects such as anxiety and depression. These scores are analysed in the course of the secondary outcomes.

Elimimian et al. (2020) [4] measured the quality of life at baseline, 12 months, and 24 months after completion of the intervention. The authors used the 36-SF survey method. The mean value of the mental component scale (MCS) at baseline was 43.7, after 12 months 46.1, and after 24 months 47.02. This corresponds to a change of 10% from baseline to 12 months after and a change of 14% from 12 months to 24 months after completion, which was statistically significant. The scores of the physical component scale (PCS) were 45.75 at baseline, 46.02 after 12 months, and 45.65 after 24 months. A 2% change from baseline to 12 months after and a 1% change from 12 months to 24 months after was noted, which the authors classified as no significant improvement [4].

The aim of the study by Lengacher et al. (2019) [6] was to investigate the effects of MBSR on the stress hormone cortisol and the pro-inflammatory cytokine interleukin-6 (IL-6). In one of their hypotheses, the authors claimed an association between reduced levels of cortisol and IL-6, and improved quality of life, measured as an increased score in the 36-SF survey. Measurements were performed at baseline and six weeks after the completion of a MBSR course. The results show a correlation of decreased IL-6 and an increased quality of life, manifesting in the subscales of physical functioning, vitality, social functioning, general health, physical health, and pain after six weeks. No correlation could be found between cortisol and an improvement in quality of life. The results suggest a decrease of IL-6 six weeks after the participation in a MBSR course, which also manifests itself in improved quality of life for that timeframe [6].

Chang et al., (2022) [5] used the EQ-5D tool for their quality of life-analysis. Measurements were performed at baseline and directly after the end of the six-week MBSR course. The authors did not find a significant change in the quality of life after the completion of the MBSR program in comparison to the measurement at baseline. As a reason for the missing effect, the authors attribute the duration of MBSR training being too short (here, six weeks rather than the usual minimum eight weeks) due to convenience for the participants and postulate that a longer intervention period could have improved the quality of life [5].

The systematic review performed by Zhang et al. (2018) [9] analysed six studies with a total of 1024 patients and investigated the connections of MBSR and quality of life. The performed heterogeneity analysis displayed substantial heterogeneity across the included studies and, as a result, a random-effects model was chosen. No significant difference between the intervention with MBSR and the control groups could be detected in this meta-analysis [9].

In their systematic review, Chang et al. (2020) [8] found that there was no significant change in the quality of life after the completion of MBSR training compared to usual care. These results are in line with the systematic review by Zhang et al. (2018) [9] in which the authors state that MBSR training can relieve stress and restlessness in short-timeframes but is not sufficient for an overall change in quality of life [8].

The results of the primary outcome (quality of life) are displayed below (Table 10).

### 3.2. Secondary Outcomes: Anxiety and Depression

Elimimian et al. (2020) [4] used the GAD-7 to evaluate anxiety in their study. By performing measurements at baseline, one year, and two years after the intervention, the authors give credit to long-term effects of MBSR on reduced anxiety. At baseline, the mean-score was 7.3, which is classified as “mild anxiety”. After one year, the score dropped to 4.92, which is classified between “minimal anxiety” and “mild anxiety”, which is a significant reduction in anxiety, and after 24 months the score decreased even further to 4.88 [4].

Lengacher et al. (2019) [6] used the STAI for their study on anxiety. Measurements were performed at baseline and after six weeks. Their results show no correlation between the decreased stress-related biomarkers of Cortisol and IL-6 and decreased scores in the anxiety measuring tool [6].

Chang et al. (2022) [5] used the DASS-21 tool, whereby their results from the anxiety-related investigation showed a mean score of 28.54 at baseline, which is classified as “severe anxiety”. After six weeks the mean score of the MBSR group decreased to 17.62, which the authors describe as a significant change [5].

Mirmahmoodi et al. (2020) [7] showed in their study that the BAI score of the intervention group decreased from 31.18 to 23.50, indicating a better outcome for anxiety levels, whereas it increased from 25.41 to 35 in the control group. The measurements were taken at baseline and eight weeks after the completion of a MBSR course [7].

The meta-analysis by Zhang et al. (2018) [9] included data from eight studies with a total of 1162 patients. The authors used a random-effects model because of the substantial heterogeneity between the studies and found that MBSR training had positive effects on anxiety compared to the control groups. They found this difference to be statistically significant [9], whereas Chang et al. (2020) [8] found no significant change of anxiety scores in their meta-analysis [8]. The anxiety impact outcomes are briefly summarised below (Table 11).

Elimimian et al. (2020) [4] used the PHQ-9 tool to collect data on the levels of depression of their participants. At baseline, the mean score was 7.62 (mild depression), 12 months after the intervention it decreased to 5.0 (minimal depression) and 24 months after the MBSR course it decreased further to 4.52 (minimal depression). The authors claim the reduction of depression scores is significant [4].

Lengacher et al. (2019) [6] could not find a reduction of depression scores in correlation with Cortisol or IL-6. For their measurement of depression, they used the CES-D tool and measurements were performed at baseline and after six weeks.

Chang et al. (2022) [5] used the DASS-21 tool to measure depression together with anxiety and stress. The authors claim that the depression scores were not improved six weeks after the intervention in comparison to the control group.

Mirmahmoodi et al. (2020) [7] measured their depression scores via the BDI-II tool. The intervention group had a mean depression score of 29 (moderate depression) at baseline and score of 17.18 (mild depression) after eight weeks. The control group started at a mean depression score of 21.04 (between mild and moderate depression), whereby their score increased to 21.59 (between mild and moderate depression) after eight weeks. Because the scores at baseline differed between the intervention and control groups, the authors performed an ANOVA test that revealed the difference between the two groups was significant [7].

Chang et al. (2020) [8] compared three studies with a total of 720 participants, in order to evaluate the correlation of MBSR training and depression. Their results show no significant improvement of depression scores in the MBSR group in comparison to the control-group [8]. 

Zhang et al. (2018) [9] used nine studies with a total of 1196 participants in their meta-analysis and found an improvement of depression scores in the MBSR group compared to the control group. The authors claim their results are significant, after they had accounted for severe heterogeneity of the studies [9].

The results of the depression impact outcomes are summarised below (Table 12).

## 4. Discussion

The primary aim of this literature analysis was to find a relation between MBSR interventions and improvements in the quality of life for women that had been diagnosed with breast cancer.

From the six studies that were included in this review, only five yielded outcomes related to quality of life, but all six studies defined anxiety and depression as either primary or secondary outcomes. The studies were included as they were published after 2018, whereby this review shall serve as an up-to-date detailed meta-analysis following that of Schell and Monsef in 2019 [3]. Thus, a big part of this discussion section will compare the results of these recent studies with the integrated findings of Schell and Monsef (2019) [3].

Only the studies by Elimimian et al. (2020) [4] and Lengacher et al. (2019) [6] showed a significantly changed score in quality of life after the intervention. All the studies provided information on the quality of life right after the completion of the MBSR intervention, but only the included study by Elimimian et al. (2020) [4] captured information on the long-term effects of MBSR by gathering data 24 months after the intervention was terminated.

The meta-analysis by Schell and Monsef (2019) [3] gathered data at an early stage (right after the intervention), medium stage (six months after baseline), and long-term stage (12 months) [3].

The data by Schell and Monsef (2019) [3] suggested a positive correlation between MBSR training and quality of life at an early stage, but no improvement in quality of life in medium, or long-term stages. Only Elimimian et al. (2020) [4] performed an evaluation of the data after 24 months. In their study, Elimimian et al. (2020) [4] showed an increase in quality of life after 12 months, which could also be detected after 24 months. These findings implied that MBSR training could improve the quality of life over a longer period. The only other study included that suggested a positive correlation between MBSR training and quality of life is the study by Lengacher et al. (2019) [6]. In this study, an improvement in quality of life in the respective intervention group was observed six weeks after the MBSR intervention. The findings of Lengacher et al. (2019) [6] for the respective timepoint (six weeks) support the early stage results by Schell and Monsef (2019) [3].

The study by Chang et al. (2022) [5] and the meta-analyses by Zhang et al. (2018) [9] and Chang et al. (2020) [8] did not find a positive correlation between MBSR training and quality of life outcomes. Chang et al. (2022) [5] conducted their second measurement right after the completion of the intervention, which can be compared to the early stage in the meta-analysis by Schell and Monsef (2019) [3]. The results of Chang et al. (2022) [5] do not match those of Schell and Monsef (2019) [3] at the early stage. The results of the two meta-analyses are in accordance with those by Schell and Monsef (2019) [3] at a medium and a long-term stage. 

For the outcomes of anxiety and depression, all six included studies yielded results.

Only the studies by Lengacher et al. (2019) [6] and the meta-analysis by Zhang et al., (2018) [9] did not find an improvement in anxiety after the MBSR intervention. All other studies suggested a significant change in the post-intervention anxiety scores.

Schell and Monsef (2019) [3] found an improvement of anxiety scores in early and medium stages, but little to no significant change in the long-term stage of their research. These findings are aligned with most findings concerning anxiety in this review. Additional focus should be placed on the fact that in their meta-analysis, Schell and Monsef (2019) [3] included studies that used the GAD-7 measurement tool for the assessment of anxiety scores. This tool was also used in the study by Elimimian et al. (2020) [4] that yielded positive results for the correlation between MBSR training and anxiety scores after 12 months, which is in line with the findings of Schell and Monsef (2019) [3], and after 24 months, which contradict the findings of Schell and Monsef (2019) [3].

All these data support the trend that MBSR training can have a positive influence on anxiety scores up to 12 months after completion of the intervention. Regarding depression score impacts, the studies included do not provide a single clear outcome. This is due to the fact that the studies by Elimimian et al. (2020) [4], Mirmahmoodi et al. (2020) [7], and the meta-analysis by Zhang et al. (2018) [9] yielded results that support a positive influence from MBSR training on depression scores, whereby on the contrary, the studies by Lengacher et al. (2019) [6], Chang et al. (2022) [5], and the meta-analysis by Chang et al., (2020) [8] did not find a significant change of depression scores after the intervention with MBSR training.

The study by Schell and Monsef (2019) [3] yielded a positive influence of MBSR training on depression in early, and medium stages, but little to no difference in the depression scores in the long-term stage. These findings resemble the findings on anxiety scores [3].

In summary, no correlating trend between MBSR training and depression can be confirmed due to the heterogeneity of these findings.

## 5. Conclusions

The aim of this review was to elucidate the impact of MBSR on quality of life, anxiety, and depression of women that had been diagnosed with breast cancer. A systematic literature search yielded six studies that fit the inclusion criteria. Two of the included studies yielded positive outcomes in quality of life, but three did not find a positive correlation between the intervention and quality of life. Four out of six studies found a positive correlation between MBSR training and anxiety scores, but only half of the included studies yielded positive results for the interaction between MBSR training and depression scores. Due to the heterogeneity of the gathered information, it is difficult to give a detailed overview of the topic.

These data must be regarded in the light of some limitations:

(1) The duration of the intervention varied between the studies included and did not always last the mandatory eight-week time period of MBSR training, which may have had an adverse impact on the effectiveness of the interventions.
(2) The number of participants in every study was less than 100, which makes it difficult to draw general conclusions about the general population.
(3) Hardly any long-term data were collected in the studies included, which rules out the projection of long-term trends.

Due to the above-mentioned limitations, further studies with larger sample sizes and longer observation periods should be conducted to provide a more detailed overview of the possible beneficial effects of MBSR training in the clinical field.

## Figures and Tables

**Table 1:** Inclusion and Exclusion Criteria.

Category	Inclusion Criteria	Exclusion Criteria
Population	Women over the age of 18, who have been diagnosed with breast cancer, who have no initial psychological co-morbidity and who undergo MBSR	Women younger than 18; women with any psychological illness diagnosed before their breast cancer diagnosis
Intervention	Any form of mindfulness-based stress reduction or mindfulness-based cognitive therapy	Any other form of psychological therapy
Control	Women suffering from breast cancer that did not receive any form of psychological therapy	Any other forms of therapy beside MBSR
Outcomes	quality of life anxiety depression	Other outcomes that cannot be allocated to the intervention
Study design	randomised controlled trials non-randomised controlled trials retrospective studies prospective studies case–control studies meta-analyses systematic reviews	Any other study design
Publication date	After 2018	Before 2018
Language	German or English	Any other language
Duration	Any duration	No limitations

**Table 2:** *Search Strategy; 6 March 2022 14:05* via *PubMed*.

Search	Query	Results
#1	**breast cancer [Title/Abstract]**	307,817
#2	**breast neoplasm [Title/Abstract]**	1013
#3	**“Breast Neoplasms”[Mesh]** Sort by: **Most Recent**	321,610
#4	**#1 OR #2 OR #3**	410,079
#5	**mindfulness based stress reduction [Title/Abstract]**	1189
#6	**#5 OR #6 OR #7**	1796
#7	**#4 AND #8**	114
#8	**(“german”[Language]) OR (“english”[Language])**	29,884,822
#9	**#9 AND #10**	112
#10	**(“2018/01/01”[Date-Publication]: “3000”[Date-Publication])**	5,816,422
#11	**quality of life**	473,503
#12	#10 AND #11 AND #12	15

**Table 3:** *Extracted items*.

	Type of Study	Number of Participants (*n*)	Primary Outcome	Secondary Outcome	Duration of the Intervention	Number of Included Studies	(Mean) Age	Stage of Disease	Treatment	MBSR Intervention Time Period
Elimimian (2020) [4]	nRCT	*n* = 94	Quality of life	Anxiety, Depression	Eight weeks	/	54.4 (SD = 8.71)	1–3	Surgery, chemotherapy and/or radiation therapy	After treatment
Chang (2022) [5]	nRCT	*n* = 51	Female sexual function, Depression	Quality of life, Anxiety	Six weeks	/	47.77 (SD = 9.29)	0–4	Mainly hormone therapy	During treatment
Lengacher (2019) [6]	RCT	*n* = 322	Cortisol-levels, IL-6-levels	Depression, Anxiety, Quality of life, Stress	Six weeks	/	56.6 (SD = 9.7)	0–3	Lumpectomy and/or mastectomy Adjuvant radiation and/or chemotherapy	After treatment
Mirmahmoodi (2020) [7]	RCT	*n* = 44	Depression, Anxiety, Perceived Stress	/	Eight weeks	/	44.14 (SD = 11.19)	Nonmetastatic stage	Mainly chemotherapy and radiotherapy and surgery	During treatment
Chang (2020) [8]	Meta- analysis	*n* = 36–322	Depression, Anxiety	Quality of life, Fatigue, Pain	Six–eight weeks	*n* = 11	43–58 *	0–4	Mastectomy, lumpectomy, chemotherapy, radiation therapy and hormonal therapy	During, and after treatment
Zhang (2018) [9]	Meta- analysis	*n* = 1505	Quality of life, Physical function, Pain, Fatigue	Anxiety, Depression, Sleep quality	Four–eight weeks	*n* = 14	40–57 *	0–3	No information	During, and after treatment

* No information on the mean age was provided by the respective authors.

**Table 4:** *RoB 2 (modified)*.

		Lengacher (2019) [6]	Mirmahmoodi (2020) [7]
**Domain 1:** **Randomisation process**	1.1. Was the allocation sequence random?	Y	Y
	1.2. Was the allocation sequence concealed until participants were enrolled and assigned to interventions?	Y	Y
	1.3. Did baseline differences between intervention groups suggest a problem with the randomisation process?	N	N
	**Risk of bias judgement**	Low	Low
**Domain 2:** **Deviations from the intended interventions (effect of assignment to intervention)**	2.1. Were participants aware of their assigned intervention during the trial?	N	N
	2.2. Were carers and people delivering the interventions aware of participants assigned intervention during the trial?	Y	Y
	2.3. If Y/PY/NI to 2.1 or 2.2: Were there deviations from the intended intervention that arose because of the trial context?	N	N
	2.4. If Y/PY to 2.3: Were these deviations likely to have affected the outcome?	/	/
	2.5. If Y/PY/NI to 2.4: Were these deviations from intended intervention balanced between groups?	/	/
	2.6. Was an appropriate analysis used to estimate the effect of assignment to intervention?	Y	Y
	2.7. If N/PN/NI to 2.6: Was there potential for a substantial impact (on the result) of the failure to analyse participants in the group to which they were randomised?	/	/
	**Risk of bias judgement**	Low	Low
**Domain 3:** **Risk of bias due to missing outcome data**	3.1. Were data for this outcome available for all, or nearly all, participants randomised?	Y	Y
	3.2 If N/PN/NI to 3.1: Is there evidence that the result was not biased by missing outcome data?	/	/
	3.3. If N/PN to 3.2: Could missingness in the outcome depend on its true value?	/	/
	3.4 If Y/PY/NI to 3.3: Is it likely that missingness in the outcome depended on its true value?	/	/
	**Risk of bias judgement**	Low	Low
**Domain 4:** **Risk of bias** **in measurement of the outcome**	4.1. Was the method of measuring the outcome inappropriate?	N	N
	4.2. Could measurement or ascertainment of the outcome have differed between intervention groups?	N	N
	4.3. If N/PN/NI to 4.1 and 4.2: Were outcome assessors aware of the intervention received by study participants?	Y	N
	4.4. If Y/PY/NI to 4.3: Could assessment of the outcome have been influenced by knowledge of intervention received?	Y	N
	4.5. If Y/PY/NI to 4.4: Is it likely that assessment of the outcome was influenced by knowledge of intervention received?	N	/
	**Risk of bias judgement**	Some concerns	Low
**Domain 5:** **Selection of the reported result**	5.1. Were the data that produced this result analysed in accordance with a pre-specified analysis plan that was finalised before unblinded outcome data were available for analysis?	Y	Y
	Is the numerical result being assessed likely to have been selected on the basis of the results from …		
	5.2. multiple eligible outcome measurements (e.g., scales, definitions, timepoints) within the outcome domain?	N	N
	5.3. multiple eligible analyses of the data?	N	N
	**Risk of bias judgement**	Low	Low
**Overall Risk of Bias**		Some concerns	Low

**Table 5:** *Table ROBINS-I (modified)*.

		Elimimian (2020) [4]	Chang (2022) [5]
**Bias due to confounding**	Potential for confounding?	N	N
	Appropriate analysis method to control for all the important confounding domains?	Y	Y
	Risk of Bias	Low	Low
**Bias in selection of participants into the study**	Selection based on participants characteristic observed after the start of the intervention?	N	N
	Most participants followed from the start of the intervention?	Y	Y
	Risk of Bias	Low	Low
**Bias in classification of interventions**	Intervention groups clearly defined?	Y	Y
	Risk of Bias	Low	Low
**Bias due to deviation from intended interventions**	Deviations from intended interventions (e.g., High drop-out rate)?	N	N
	Deviation unbalanced between groups?	N	N
	Risk of bias	Low	Low
**Bias due to missing data**	Outcome data available for nearly all participants?	Y	Y
	Participants excluded due to missing data?	N	N
	Risk of Bias	Low	Low
**Bias in measurement of outcomes**	Could the outcome measure have been influenced by knowledge of the intervention received?	N	N
	Were outcome assessors aware of the intervention received by study participants?	NI	N
	Were the methods of outcome assessment comparable across intervention groups?	Y	Y
	Risk of Bias	Moderate	Low
**Bias in selection of the reported result**	Results likely to be selected from multiple measurements?	N	N
	Risk of bias	Low	Low
**Randomisation**		N	N
**Overall risk of bias**		Moderate	Low

**Table 6:** *GAD-7 Scores* [4].

Test Score	Result
0–4	Minimal Anxiety
5–9	Mild Anxiety
10–14	Moderate Anxiety
15–21	Severe Anxiety

**Table 7:** *PHQ9 Scores* [4].

Test Score	Result
0–4	Minimal Depressive Symptoms
5–9	Mild Depressive Symptoms
10–14	Moderate Depressive Symptoms
15–27	Severe Depressive Symptoms

**Table 8:** *DASS-21 Scores* [5].

	Depression	Anxiety	Stress
Normal	0–4	0–3	0–7
Mild	5–6	4–5	8–9
Moderate	7–10	6–7	10–12
Severe	11–13	8–9	13–16
Extremely Severe	>14	>10	>17

**Table 9:** *BAI Scores* [15].

Test Score	Result
0–21	Mild Anxiety
22–35	Moderate Anxiety
>36	Severe Anxiety

**Table 10:** *Primary Outcome (Quality of Life) Results*.

	Measured (via)		Significant Change
Elimimian et al. (2020) [4]	36-SF	MCS-subscore	Yes
		PCS-subscore	No
Lengacher et al. (2017) [6]	36-SF	Cortisol	No
		IL-6	Yes
Chang et al. (2022) [5]	EQ-5D		No
Zhang et al. (2018) [9]	Meta-analysis		No
Chang et al. (2020) [8]	Meta-analysis		No

**Table 11:** *Anxiety Outcomes*.

	Measured (via)	Significant Change
Elimimian et al. (2020) [4]	GAD-7	Yes
Lengacher et al. (2019) [6]	STAI	No
Chang et al. (2022) [5]	DASS-21	Yes
Mirmahmoodi et al. (2020) [7]	BAI	Yes
Chang et al. (2020) [8]	Meta Analysis	Yes
Zhang et al. (2018) [9]	Meta Analysis	No

**Table 12:** *Depression Outcomes*.

	Measured (Via)	Significant Change
Elimimian et al. (2020) [4]	PHQ-9	Yes
Lengacher et al. (2019) [6]	CES-D	No
Chang et al. (2022) [5]	DASS-21	No
Mirmahmoodi et al. (2020) [7]	BDI-II	Yes
Chang et al. (2020) [8]	Metanalysis	No
Zhang et al. (2018) [9]	Metanalysis	Yes
